# Animal model of a bovine pericardial patch for thoracoabdominal aortic aneurysms: step by step

**DOI:** 10.1590/1677-5449.202401822

**Published:** 2025-06-13

**Authors:** Allana Maryel Tobita, Bruno Jeronimo Ponte, Maria Fernanda Cassino Portugal, Anna Paula Weinhardt Baptista, Igor Rafael Sincos, Nelson Wolosker

**Affiliations:** 1 Hospital Israelita Albert Einstein, São Paulo, SP, Brasil.; 2 Universidade de São Paulo, Faculdade de Medicina, São Paulo, SP, Brasil.

**Keywords:** thoracoabdominal, aortic aneurysm, animal model, toracoabdominal, aneurisma aórtico, modelo animal

## Abstract

**Background:**

The treatment of thoracoabdominal aortic aneurysms (TAA) has advanced. Understanding the pathophysiology and surgical approaches to this disease is essential for best therapeutic performance.

**Objectives:**

We aimed to improve previously described methods for creating thoracoabdominal aortic aneurysms in a porcine animal model, reducing surgical procedure time and specimen mortality.

**Methods:**

A total of 18 swine underwent a surgical procedure to create a TAA. An autologous peritoneal patch was used to create the aneurysm in 2 animals, and a bovine pericardial patch was used in the other 16. The animals were followed up postoperatively, and the aneurysm sac was reexamined after 4 weeks. The animals that did not die in the post-operative period were euthanized according to institutional recommendations.

**Results:**

All of the animals underwent laparotomy with retroperitoneal access. Two received an autologous peritoneal patch and 16 received a bovine pericardial patch. Three animals underwent single suprarenal clamping, while 15 underwent sequential clamping. There were no differences in operative time (p*=*0.207) or total clamping time (p*=*0.276) between groups. There was a higher mortality rate after 4 weeks in animals that received single clamping (100%) than sequential clamping (26.7%) (p*=*0.0017).

**Conclusions:**

The experimental model of TAA using a bovine pericardial patch and a sequential clamping technique provided a stable and reliable platform that remains stable and patent for up to 4 weeks. This model can be extremely valuable for assessing new endovascular therapy options in living organisms.

## Resumo

**Contexto:**

O tratamento dos aneurismas da aorta toracoabdominal (ATA) tem avançado. A compreensão da fisiopatologia e das abordagens cirúrgicas dessa doença é essencial para o melhor desempenho terapêutico.

**Objetivos:**

Descrever e otimizar os métodos previamente estabelecidos para a confecção de ATA em modelos animais suínos, objetivando reduzir o tempo do procedimento cirúrgico e a mortalidade das amostras.

**Métodos:**

Dezoito suínos foram submetidos a procedimento cirúrgico para confecção de ATA. O aneurisma foi confeccionado utilizando retalho de peritônio autólogo em dois animais e retalho de pericárdio bovino em 16. Os animais foram monitorados no pós-operatório e reabordados após 4 semanas para análise do saco aneurismático. Os modelos que sobreviveram ao pós-operatório foram submetidos à eutanásia, conforme as recomendações institucionais.

**Resultados:**

Todos os animais foram submetidos à laparotomia com acesso retroperitoneal. Dois receberam *patch* de peritônio autólogo, enquanto 16 *patch* de pericárdio bovino. Três modelos foram submetidos a clampeamento suprarrenal único, enquanto 15 passaram por clampeamento sequencial em duas etapas. Não houve diferenças significativas no tempo cirúrgico (p *=* 0,207) ou no tempo total de clampeamento (p *=* 0,276) entre os grupos. Observou-se maior mortalidade após 4 semanas nos modelos submetidos a pinçamento único (100%) em relação com o pinçamento sequencial (26,7%) (p *=* 0,0017).

**Conclusões:**

O modelo experimental de ATA com pericárdio bovino, utilizando a técnica de clampeamento sequencial, demonstrou ser estável e confiável, com anatomia e patência consistentes até 4 semanas. Este modelo pode ser valioso para novas opções terapêuticas endovasculares em organismos vivos.

## INTRODUCTION

Over the years, experimental models have been crucial in deepening our understanding of the pathophysiology of various diseases and have played a major role in the search for effective therapeutic interventions. Animal experimental trials are particularly important in biomedical fields where applicable *in vitro* models are limited. Although these studies are subject to strict legal and ethical restrictions, they are essential for developing safe protocols that can be directly applied to human patients. They represent an important stage in evaluating the efficacy of new medical devices and pharmacological therapy before their application in clinical trials.^1,2^

Despite advancements in surgical techniques, diagnosing and treating diseases that affect the thoracoabdominal aorta, such as aneurysms and dissections, remains challenging. Experimental models are crucial for evaluating disease progression and testing new therapies in this field.^3-9^

Several groups have described animal models for studying thoracoabdominal pathologies, although with significant variation.^10^ However, the invasiveness of surgical models has been limited, and it is challenging to establish a proper intensive care system for the high morbidity and mortality associated with procedures of this magnitude.

Our experience with experimental porcine models has helped us develop procedures to improve animal handling and reduce complications, especially for thoracoabdominal interventions.^8,9^ We have recently created a protocol to study the hemodynamic effects of the Multilayer Flow Modulator Stent (Cardiatis, Isnes, Belgium) in artificially induced thoracoabdominal aneurysms in porcine models.^9^ This latest study represents the culmination of our work and serves as a refinement of practices that led to these procedural standards.

This report outlines our procedures to assist other researchers in developing protocols for implementing an experimental animal model for thoracoabdominal aneurysms with minimal complications and animal loss.

## METHODS

The study was conducted between November 2016 and April 2019 at the Center for Experimentation and Training in Surgery of a quaternary hospital in São Paulo, Brazil. This center has been accredited by the Association for Assessment and Accreditation of Laboratory Animal Care and Use Committee. The completed Animals in Research: Reporting *in vivo* Experiments (ARRIVE) checklist is shown in the Appendix A. The protocol was approved by our institution’s Animal Use Ethics Committee (number 3312/2018). The ARRIVE guidelines were duly respected and followed.

### Animal selection and preoperative anesthetic procedures

Eighteen Large White pigs, aged 4 to 10 months and weighing 37 to 75 kg, were selected. No animal was excluded from the study protocol. The animals were raised and cared for at the experimental surgery center of a quaternary hospital.

Before surgery, food and water were withheld from the pigs for 12 hours. The protocol required pre-anesthesia with ketamine (10.0 mg/kg) and midazolam (0.25 mg/kg), which were administered intramuscularly.

A 22-gauge BD Insyte catheter (BD Infusion Therapy Systems Inc., Sandy, UT, USA) was used to catheterize the marginal ear vein for venous access. The right carotid artery was catheterized to measure invasive arterial pressure.

To induce anesthesia, etomidate (1 mg/kg) and propofol (5 mg/kg) were administered. Size 7.0 Portex endotracheal tubes (Smiths Medical, Ashford, UK) were used for intubation. For inhalational anesthesia, 1.5% isoflurane was used with the ventilator set at a tidal volume of 10 mL/kg. Fentanyl (2.5 mg/kg) was used to maintain anesthesia.

Fluid was maintained using a crystalloid solution at a rate of 10 mL/kg/h. Crystalloid solution was also used at a rate of 1 to 2 mL/kg/h as needed in bolus form to maintain a mean blood pressure of ≥ 70 mm Hg. According to protocol, animals with unresponsive hypotension were to be excluded from the study.

All animals received antibiotic prophylaxis with benzathine penicillin G (2.4 million units IM) and cephazolin (1 g IV).

### Intraoperative technique

The initial protocol reproduced Maynard’s technique, where an aneurysmal sac was created using the native peritoneum.^10^ The aneurysmal patch was made of peritoneal tissue during surgery in the first two animals. However, this technique prolonged the operation and had hemodynamic consequences, so it was abandoned for the following animals.

### Aneurysmal patch creation

**Figure 1:**
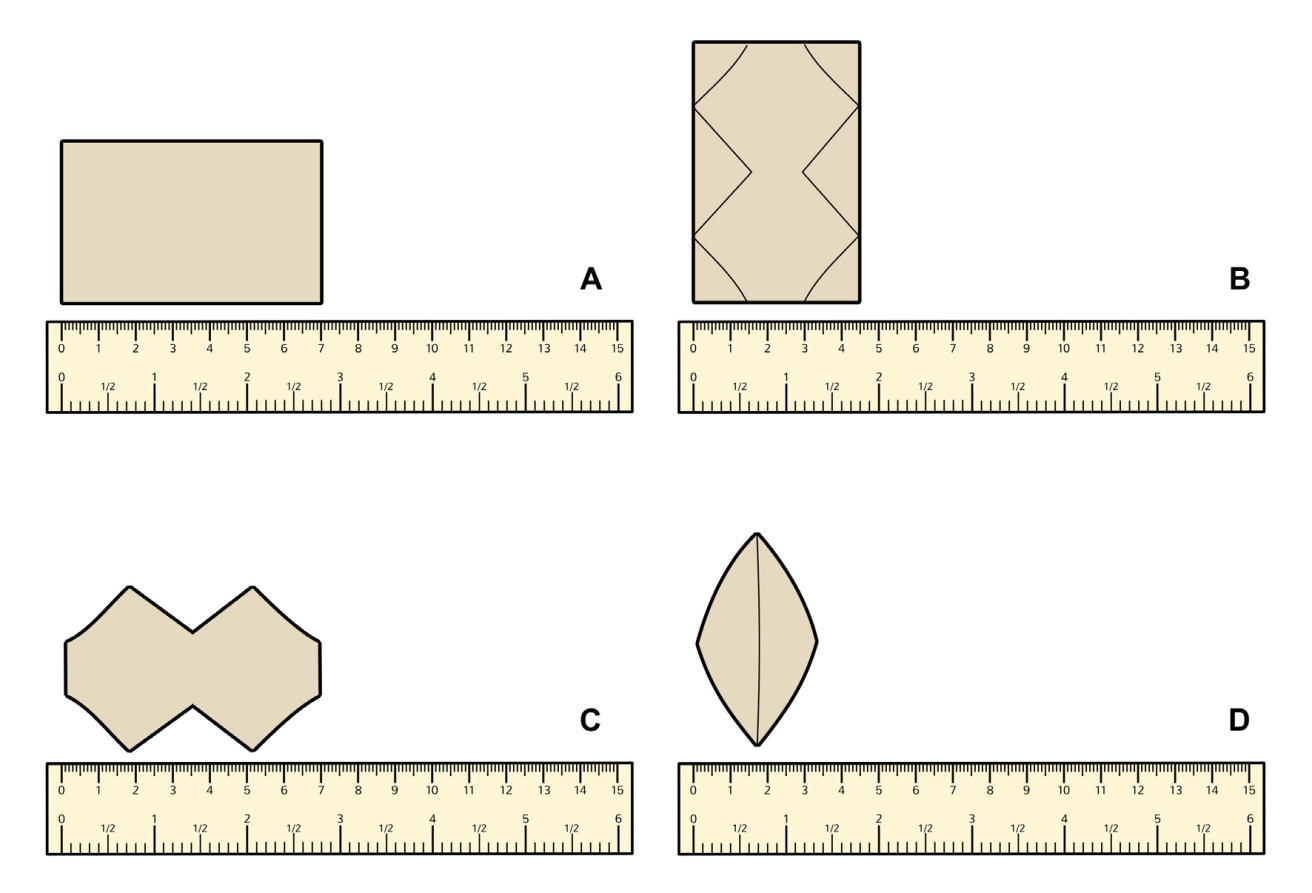
(A) The bovine pericardial patch; (B) The trapezoid shape being drawn on the pericardial patch; (C) Two trapezoid-shaped leaflets were made; (D) The trapezoid-shaped pericardium was molded into an oval configuration after a continuous suture using Prolene 5-0.

In 16 cases, the aneurysmal patches were prepared on a secondary table prior to the procedure. A bovine pericardial patch (Braile Biomedica, São José do Rio Preto, São Paulo) was folded in half, and a trapezoid pattern was drawn onto the patch using a surgical marker (Figure 1). It was then molded into two equal trapezoid leaflets, which were sutured together in an oval configuration using a continuous Prolene 5-0 suture. The approximate final dimensions of the patch were 7 cm (length) x 4.5cm (width).

### Surgical access and aneurysm creation

All animals underwent laparotomy with retroperitoneal dissection to access the visceral aorta, which was controlled at the top (at the level of superior mesenteric artery) and the bottom (at the iliac bifurcation level). The renal and lumbar arteries were also identified and preserved during the procedure.

In all animals, a systemic dose of heparin at 200 UI/kg was administered 2 minutes before the proximal and distal clamping of the aorta.

### Clamping type and intraoperative technique

Single clamping: In the first 3 animals, the aorta was clamped to the renal arteries simultaneously, which resulted in a longer suprarenal clamping time than sequential clamping and significantly impaired the intraoperative hemodynamic balance. As a result, this approach was abandoned beginning with the fourth animal.

Two-step sequential clamping: In 15 animals, a clamp was placed on the aorta just below the renal arteries. Once the clamp was secure, a 6 cm incision was made just below the emergence of the renal arteries, and a thin elliptical patch of the aortic wall measuring 3 mm was removed. The aneurysmal patch, made of bovine pericardium, was then attached to the incision with a continuous Prolene 4-0 suture.

**Figure 2:**
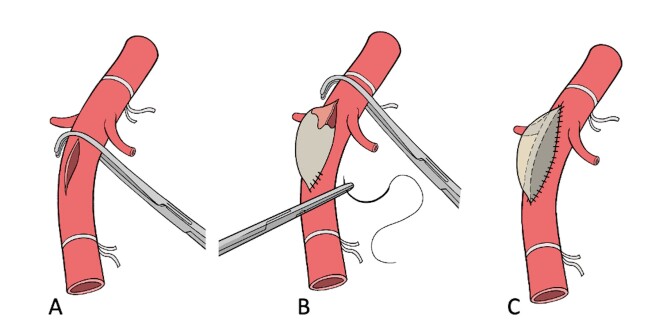
Two-step sequential clamping: (A) Initially, the clamp is placed under the renal artery. A 6 cm incision is made and the distal part of the patch is sutured at the aortic wall; (B) The clamp is then moved 1 cm above the renal artery; (C) completing the suture and concluding the creation of the aneurismal sac.

**Figure 3:**
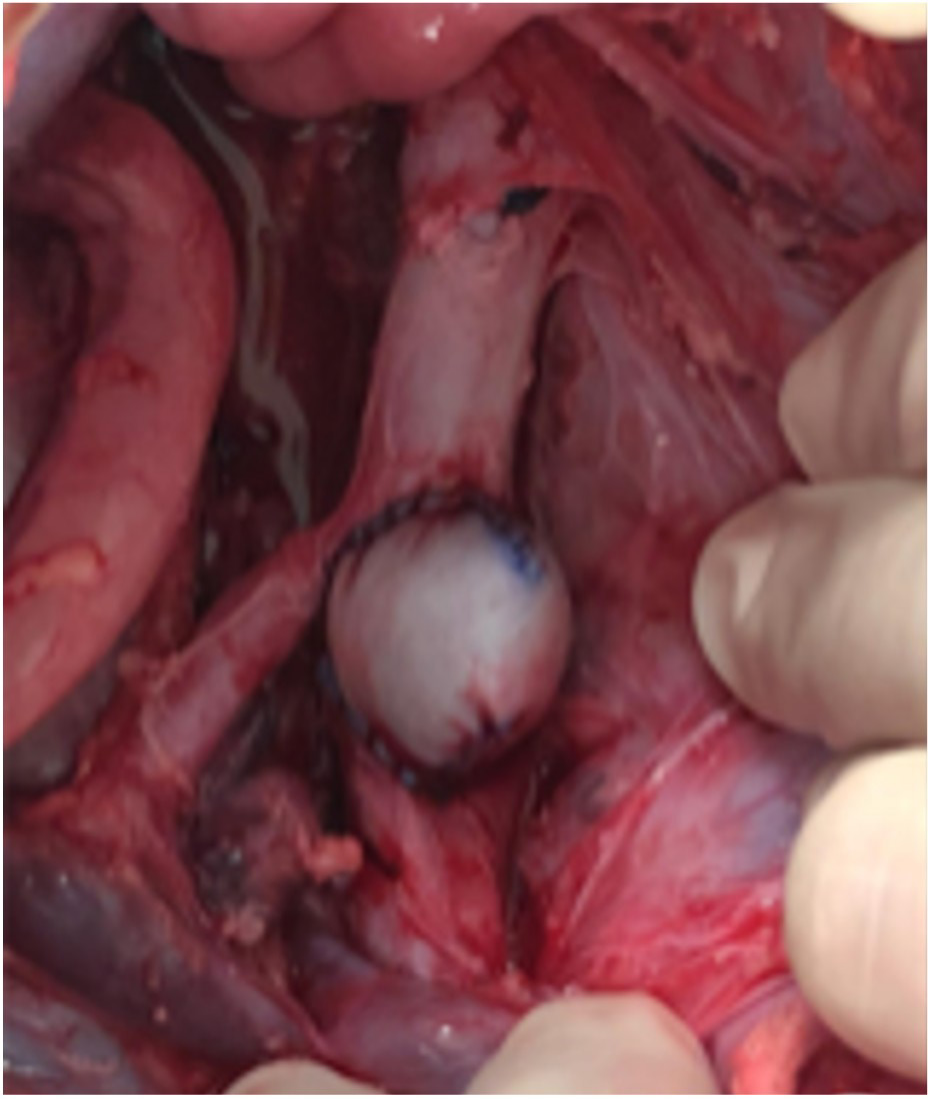
Final aspect of the saccular aneurysm.

The clamp was then moved 1 cm upward along the aorta (second step), above the renal artery ostium. The incision was then extended, and the proximal part of the patch was sutured to the aorta at the level of the renal arteries, thus completing the aneurysmal sac. The two-step sequential clamping technique is illustrated in Figure 2, and the final aspect of the saccular aneurysm is shown in Figure 3.

After careful review, aortic flow was restored, and the renal and lumbar arteries were unclamped.

### Hemodynamic and electrolytic assessment

Blood samples were collected before aortic clamping and whenever needed during the procedure and tested using an i-STAT analysis system (Abbott Point-of-Care, East Windsor, NJ, USA).

### Intraoperative imaging control and assessment of technical success

**Figure 4:**
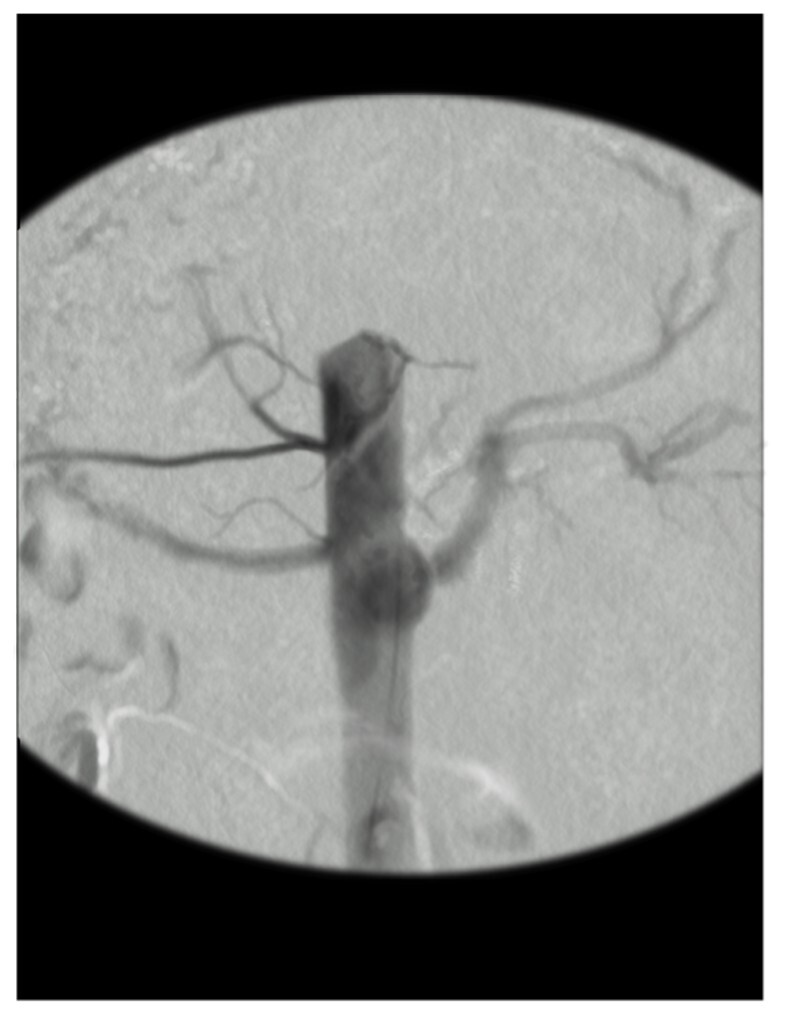
Final aspect after aortography, demonstrating the creation of a saccular aneurysm.

After restoring blood flow, intraoperative aortography was performed using a 5F pigtail catheter (Impulse, Boston Scientific, Marlborough, MA, USA) with radiopaque centimeter markings positioned at the level of the first lumber vertebrae and inserted through the femoral artery. We measured the diameter and extent of the aneurysm and the healthy aorta (Figure 4).

We considered cases in which the aneurysmal section was enlarged to ≥ 50% of the diameter of the healthy aorta to be technical successes.

### Postoperative protocol

To prevent intestinal slowing, solid food was withheld for 24-48 h after surgery. Water was provided *ad libitum* 24 h postoperatively. When the animals started eating again, they were given dog food (Hill’s Critical Care, Hill's Pet Nutrition, Inc.) until they fully accepted their normal dry diet.

Fluid therapy began on the day before surgery with a solution containing NaCl 0.9% 1000 ml + 50% glucose 100 ml every 8h, given every 8 h for the first 48 h and was adjusted as needed.

Following veterinary determinations, the pigs were given daily medication to decrease stomach acid production (ranitidine 2 mg/Kg, intravenously or intramuscularly, thrice daily), and when necessary, an antiemetic (metoclopramide 0.5 mg/Kg, intravenously or intramuscularly up to three times daily), and pain relief (ketoprofen 5mL intravenously, once daily and dipyrone 25 mg/Kg intravenously or intramuscularly, up to twice daily). Opioids were only used in the first 2 days after surgery (morphine 30 mg intravenously, twice daily).

Antibiotics (cefazolin 1 g, intravenously or intramuscularly) were maintained for 5 days postoperatively. Wound cleaning and dressing were performed twice daily with dexamethasone, neomycin, nystatin, and benzocaine cream.

### Reassessment

After 4 weeks, the animals underwent a second procedure involving laparotomy. They were assessed for vessel patency, patch infection or rupture, and leaks. The follow-up procedure involved the same anesthetic and surgical protocols used in the initial procedure.

**Figure 5:**
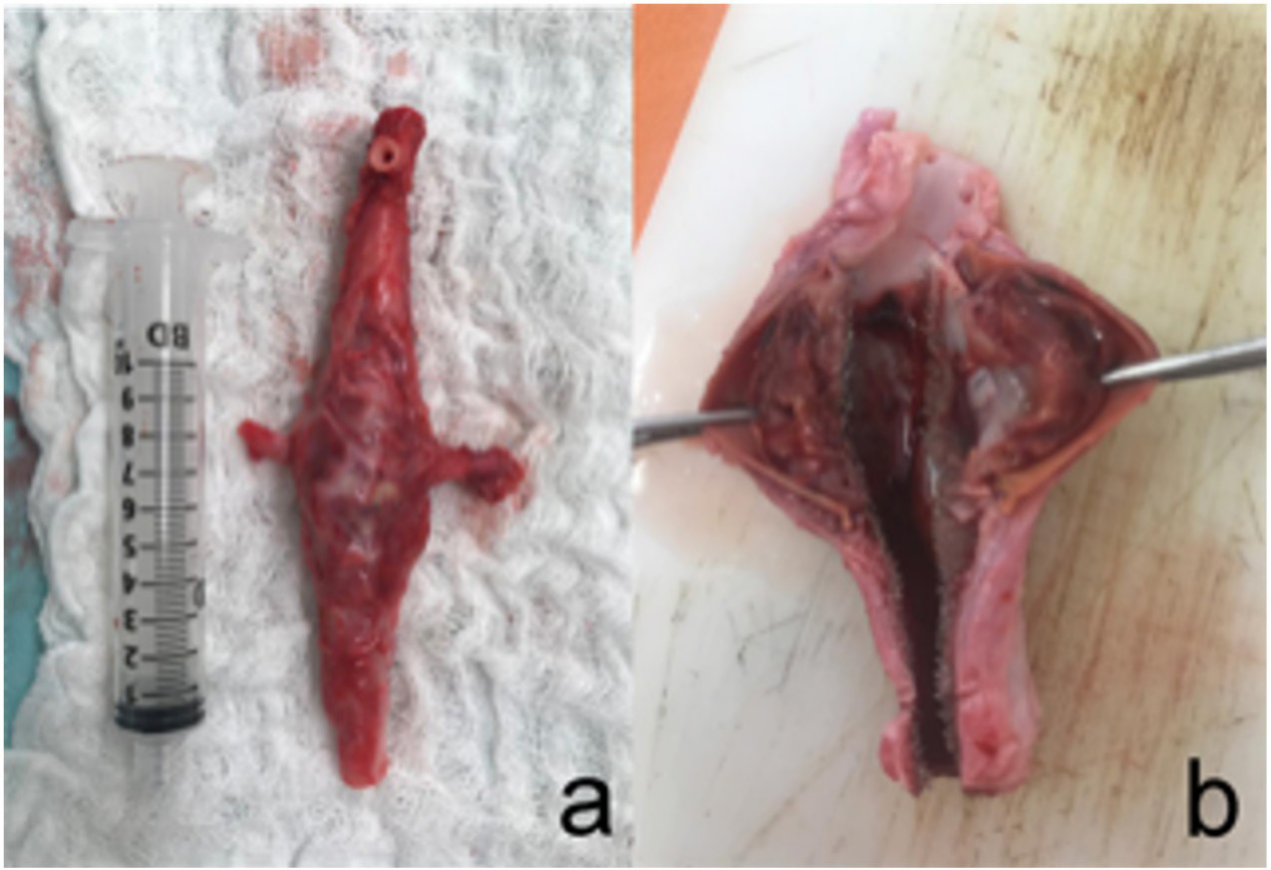
(A) Aspect after explantation of the aneurysmal aorta; (B) Morphological aspect of the aneurysm sac.

Immediately after reassessment and while still under general anesthesia, all animals were euthanized with a KCl solution. Aortic explantation was performed for morphological analysis (Figure 5). The animals were observed for 4 weeks, with a mean weight gain of 12.06 (SD, 8.09) Kg.

### Statistical analysis

Categorical data were expressed as absolute frequencies and percentages, while continuous data were expressed as means with SD and minimum-maximum values. Associations between categorical data were investigated using Fisher’s exact test, while continuous data were analyzed using the Mann-Whitney test.

Linear models were adjusted to assess the effects of each technique on each group. The results are presented as mean-adjusted values with standard errors and 95% CI. The *p*-values were obtained from multiple comparisons between measurements and group procedures.

All analyses were performed in IBM SPSS Statistics 19.0 (IBM Corp, Armonk, NY), with p < 0.05 considered significant.

## Results

### Primary procedure

The success rate of the the primary procedure for aneurysm creation was 100%, and the survival rate was 88.88%. The mean operative time was 176.22 (SD, 40.96) minutes (range 105-255 min). When the technique for creating the aneurysmal sac from the native peritoneum was replaced with previously prepared bovine pericardium, the mean operative time was reduced to 171.87 (SD, 38.24) min (range 105-170 min), which, although notable, was not statistically significant (*p* = 0.207).

### Outcomes according to clamping type

Eighteen animals were included in the study. Three animals underwent single clamping and 15 underwent two-step sequential clamping (Table 1). There were no significant differences in operative time (p=0.207) or total clamping time (p=0.276) between groups. However, there was a significant reduction in suprarenal clamping time (p = 0.05), with a mean reduction of > 30 minutes.

**Table 1:** Outcomes of the single clamp and two-step sequential clamp techniques.

Group	Single Clamp (n = 3)	Two-Step Clamp (n=15)	*p-value*
Total operative time (min)	210.66 ± 44	169.33 ± 38.1	0.207
Total clamping time (min)	46.66 ± 12.58	27.26 ± 14.45	0.276
Suprarenal clamping time (min)	46.66 ± 12.58	12.06 ± 6.55	0.05
All-cause mortality (%)	100	26.7	0.017

There was a significant association between the single clamp technique and death before 4 weeks (p=0.017). Sequential clamping resulted in a 73% mortality reduction in this group. All animals (100%) in the single clamping group died, while in the sequential clamping group, only 4 (26.7%) animals died before the 4-week follow-up.

### Follow-up and study protocol completion

After four weeks, all of the surviving animals had patent aneurysms. There were no ruptures, infections, or occlusions in the aorta or its branches. The animal protocol completion rate was 61.1%. Adverse outcomes are shown in Table 2 and discussed below. After euthanasia, aortic morphological analysis showed patent aneurysms for all animals that did not complete the study.

**Table 2:** Individual animal outcomes.

Animal Characteristics		Outcome	Survived until 4-week reevaluation
Animal study number	Aneurysm Graft		
01	peritoneal	Ischemia-reperfusion injury, death before 4 weeks	No
02	peritoneal	Paraplegia followed by euthanasia, death before 4 weeks	No
03	bovine per.	Ischemia-reperfusion injury, death before 4 weeks	No
04	bovine per.	Completed the study protocol	Yes
05	bovine per.	Intestinal occlusion, death before 4 weeks	No
06	bovine per.	Completed the study protocol	Yes
07	bovine per.	Completed the study protocol	Yes
08	bovine per.	Completed the study protocol	Yes
09	bovine per.	Completed the study protocol	Yes
10	bovine per.	Expansive cervical hematoma, immediate postop. death	No
11	bovine per.	Non-responsive hypotension, immediate postop. death	No
12	bovine per.	Paraplegia followed by euthanasia, death before 4 weeks	No
13	bovine per.	Completed the study protocol	Yes
14	bovine per.	Completed the study protocol	Yes
15	bovine per.	Completed the study protocol	Yes
16	bovine per.	Completed the study protocol	Yes
17	bovine per.	Completed the study protocol	Yes
18	bovine per.	Completed the study protocol	Yes

per.: pericardial.

## Discussion

### Animal selection

Experimental models cannot perfectly replicate human physiopathology,^11,12^ but pigs are valuable for research due to similarities in size, physiology, organ development, and disease progression.^13-15^ The immune systems of swine, particularly inbred varieties with fixed histocompatibility complexes, yield more reproducible results than other species such as dogs and sheep.^16,17^ The Large White breed has been validated for pharmacological trials, meeting size and development requirements for various protocols.^10^ The animals used in this study weighed 37-74 kg, which aligns with the range for thoracoabdominal disease trials ranges (20-67 kg).^10,18^ A minimum weight of 50 kg is ideal for surgical procedures due to robust anatomy and larger vessel diameter. However, rapid weight gain, which is limited by housing restrictions at four weeks, complicates long-term follow-up.

Our swine studies^13,14^ have demonstrated that the 50 kg minimum weight threshold is ideal for surgical procedures due to the animal’s robust constitution and larger vessel diameter. However, these animals gain weight quickly, which could be a drawback for long-term follow-up.

### Anesthetic and preoperative procedures

Preoperative medications in swine can reduce anxiety and the need for general anesthetics.^19^ Anticholinergics prevent vagal reflexes during intubation and cardiovascular manipulation.^19^ Inhalation anesthesia is preferred but requires proper equipment and monitoring. Injectable anesthetics are convenient for induction or short-term procedures, effectively complementing inhalation protocols.^19^

### Aneurysmal patch production

The aneurysmal wall was originally constructed using a patch from the animal’s own peritoneum, as suggested by Maynar et al.^10^ This technique could potentially eliminate the need for additional material while creating an autogenic aneurysmal sac. As a result, it may respond more like organic tissue and have the potential for further sac growth.^10^

The initial premise turned out to be flawed because the peritoneum graft technique exponentially increased operative time, resulting in complications such as ischemia-reperfusion syndrome and hemodynamic instability. Maynar et al. reported a mean operative time of 120 minutes, with aortic occlusion time varying from 60 to 95 minutes (78 [SD, 16] minutes). However, in their study, only 4 of 27 pigs in which an aneurysm was created survived the 60-day follow-up period.^10^ The authors attributed the deaths to several complications, including acute renal failure, intestinal obstruction, pulmonary embolism, and two from unknown causes. Two other animals were euthanized as a consequence of paraplegia and extreme weight loss. The aneurysm rupture rate in the first two weeks of follow-up was 55.5%.^10^

In our study protocol, the peritoneum patch technique was discontinued after the second case in favor of the bovine pericardial patch. This approach resulted in a notable decrease in total operative time (176.22 [SD, 40.96] to 171.87 [SD, 38.24] min; p = 0.207), although a significant correlation was not determined.

Furthermore, the previously described sequential clamping technique significantly impacted the animals’ hemodynamic patterns and electrolyte balance during both the surgical and postoperative periods. The mortality rate in the sequential clamping group (26.7%) was much lower than that of the single clamping group (100%). The high mortality rate in the single clamping group is consistent with findings from other animal studies.

Uflacker and Brothers’^20^ technique of creating an artificial patch in a saccular shape does not offer the advantage of mimicking the shape and size configurations of conventional thoracoabdominal aortic aneurysms, unlike the patch interposition model of Parodi et al.^21^ However, unlike Parodi et al.’s configuration, the saccular approach provides more accurate results on the role of the aneurysmal wall and the effects of patent aortic side branches, which are indispensable when analyzing the thoracoabdominal region.^21^

Several studies have effectively used bovine pericardial patches to create artificial aneurysms in experimental models, achieving good patency rates.^7,18,22^ These patches can expand, simulating an aneurysmal segment due to antigenic degeneration. However, because it is an acellular material, this response is minimal and does not incur high rupture rates like vein grafts or their synthetic counterparts.^23^ The literature describes patch sizes for aneurysm creation ranging from 3 cm x 3 cm to 3 cm x 6 cm in pigs and 6 cm x 5 cm x 8 cm in sheep.^22^

### Postoperative analgesia and euthanasia

After surgery, pigs may show signs of distress, such as restlessness and food refusal.^19^ Newer non-steroidal anti-inflammatory drugs have been successfully used for postoperative pain relief, either orally or by injection. Parenteral analgesics are usually administered intramuscularly or subcutaneously in the neck. Pigs can be easily encouraged to take oral medication by mixing it with their food.^19^

For euthanasia, most injectable forms used in other large animal species are suitable for pigs. Pentobarbital overdose (>150 mg/kg) is the preferred form of parenteral euthanasia.^19^ KCl injections or exsanguination may be performed while the pig is under general anaesthesia.^24^

### Protocol Outcomes

Our experimental model achieved a 100% success rate in aneurysm creation and no deaths occurred during the primary procedure. In a porcine model, Bischoff et al. reported deploying an endograft in the abdominal aorta to evaluate spinal ischemia, with the only complication being a stent migration covering the celiac axis.^25^

Okuno et al. designed an experimental porcine model to assess thoracoabdominal dissection. In the primary procedure, an artificial aortic dissection was created with a 78.6% success rate. The 3 failed cases included 1 guide wire trapped in the false lumen and 2 intraoperative aortic ruptures.^3^

Two experimental swine trials previously published by our group, assessing aorta stent-graft oversizing^13^ and the rheological effect of renal ischemia and reperfusion in pigs, also had 100% success and intraoperative survival rates.^8^

Finally, the technical success and survival rates in our trial’s primary procedure did not differ remarkably from similar artificial aortic saccular aneurysm studies. In Aquino et al.^7^ and Kalder et al.,^22^ the technical success of aneurysm creation was also 100%.

### Follow-up and protocol completion

The follow-up intervals in our trials have generally been limited due to space constraints in the Centre for Experimentation and Training in Surgery, particularly for the Large White breed, which gains weight relatively quickly in confinement.^8,25^ Monitoring these animals for an extended period would compromise their well-being due to their size and weight. Therefore, we have found that a 4-week follow-up period is typically sufficient to determine the outcomes without jeopardizing the animals’ welfare.

The bovine pericardial patches showed 100% aneurysm patency after 4 weeks. Similarly designed trials also reported 100% aneurysm patency after 2 weeks,^7^ and at the 52-week follow-up.^22^ Aquino et al. described parietal thrombus formation in all subjects, with 2 occluded (18%; 95% CI = 3.98-48.84) and 9 patent aneurysms (82%; 95% CI = 51.15-96.01).^7^

In our sample, no ruptures or aortic or branch occlusions were encountered. Kalder et al. reported 1 case of hemorrhage due to aneurysmal suture line and 1 case of infection and rupture of the aneurysmal patch.^22^

The protocol completion rate in our trial was 61.1%. Okuno et al. conducted follow-up observations in 5 of their original 14 pigs, 2 of which died before the end of the 5-day follow-up due to confirmed aortic rupture.^3^ In Kalder et al.,^22^ 2 of the 6 original animals died before the protocol was completed. The first animal died within 1 week as a consequence of aneurysmal suture line bleeding, and the second one died after 48 weeks due to infection and rupture of the aneurysmal patch. Aquino et al.,^7^ however, reported that all 11 animals in their sample completed the protocol.

Our study found that the main reasons for animal loss during follow-up were long aortic clamping intervals. According to Table 2, 7 animals were lost during follow-up, with 5 perishing due to the consequences of increased clamping time. Ischemia and reperfusion injury (n = 3) or paraplegia induced by spinal ischemia (n = 2) were the main causes of death. Additionally, there were cases of digestive tract complications and respiratory insufficiency following cervical expansive hematoma.

Sincos et al. accessed iliac arteries through a retroperitoneal approach, avoiding the need for peritoneal exposure.^13^ However, to implant the thoracoabdominal aneurysmal patch and retract both renal arteries, the aorta had to be exposed at the level of the visceral branches, which was hindered by this laborious approach. As a result, transperitoneal access with aortic clamping was used. Although these two samples were not directly compared, the late mortality rate was remarkably higher in the latter trial (0% vs 44.4%), as is commonly seen in procedures involving peritoneal exposure and suprarenal clamping.

### Limitations

While developing the protocols for our trials,^9^ we learned that a shorter operative time is paramount for minimizing postoperative complications and mortality. We shortened the operative time by using a secondary table in the operating room to create an aneurysmal patch from bovine pericardium, with two parallel teams working together.

Several factors must be considered when applying an experimental animal model. No animal model will ever perfectly mimic human pathophysiology, so it is important to use multiple models to gain a better understanding before choosing the most appropriate one. This means it is crucial to clearly define the hypothesis to develop an experimental protocol that could lead to relevant clinical data.

## CONCLUSIONS

In our swine model, a bovine pericardial patch effectively reduced the operative time. Sequential two-step clamping decreased the suprarenal clamping time and overall mortality. An experimental thoracoabdominal aortic aneurysm model involving a bovine pericardial patch and a sequential clamping technique provided a stable platform that remained patent for up to 4 weeks. This model could be extremely valuable for assessing new endovascular therapy options in living organisms.

## Appendix A Reporting checklist for study using laboratory animals. Based on the ARRIVE guidelines.

**Table:**

Essential 10		Reporting item	Page number
Study design	#1a	Give details of the groups being compared, including control groups. If no control group has been used, the rationale should be stated.	2
Study design	#1b	Give details of the experimental unit (e.g., a single animal, litter, or cage of animals).	2
Sample size	#2a	Specify the exact number of experimental units allocated to each group, and the total number in each experiment. Also indicate the total number of animals used.	2
Sample size	#2b	Explain how the sample size was decided. Provide details of any a priori sample size calculation, if done.	
Inclusion and exclusion criteria	#3a	Describe any criteria used for including or excluding animals (or experimental units) during the experiment, and data points during the analysis. Specify if these criteria were established a priori. If no criteria were set, state this explicitly.	2
Inclusion and exclusion criteria	#3b	For each experimental group, report any animals, experimental units, or data points not included in the analysis and explain why. If there were no exclusions, state so.	2
Inclusion and exclusion criteria	#3c	For each analysis, report the exact value of n in each experimental group.	2
Randomisation	#4a	State whether randomisation was used to allocate experimental units to control and treatment groups. If done, provide the method used to generate the randomisation sequence.	2
Randomisation	#4b	Describe the strategy used to minimise potential confounders such as the order of treatments and measurements, or animal/cage location. If confounders were not controlled, state this explicitly.	2
Blinding	#5	Describe who was aware of the group allocation at the different stages of the experiment (during the allocation, the conduct of the experiment, the outcome assessment, and the data analysis).	2
Outcome measures	#6a	Clearly define all outcome measures assessed (e.g., cell death, molecular markers, or behavioural changes).	2
Outcome measures	#6b	For hypothesis-testing studies, specify the primary outcome measure, i.e., the outcome measure that was used to determine the sample size.	5
Statistical methods	#7a	Provide details of the statistical methods used for each analysis, including software used.	5
Statistical methods	#7b	Describe any methods used to assess whether the data met the assumptions of the statistical approach, and what was done if the assumptions were not met.	5
Experimental animals	#8a	Provide species-appropriate details of the animals used, including species, strain and substrain, sex, age or developmental stage, and, if relevant, weight.	2
Experimental animals	#8b	Provide further relevant information on the provenance of animals, health/immune status, genetic modification status, genotype, and any previous procedures.	2
Experimental procedures	#9a	For each experimental group, including controls, describe the procedures in enough detail to allow others to replicate what was done, how it was done, and what was used.	2
Experimental procedures	#9b	Timing and frequency of procedures	2
Experimental procedures	#9c	Where procedures were carried out (including detail of any cclimatization periods).	2
Experimental procedures	#9d	Rationale for procedures	2
Results	#10a	For each experiment conducted, including independent replications, report summary/descriptive statistics for each experimental group, with a measure of variability where applicable (e.g., mean and SD, or median and range).	6
Results	#10b	If applicable, for each experiment conducted, including independent replications, report the effect size with a confidence interval.	6
Recommended set			
Abstract	#11	Provide an accurate summary of the research objectives, animal species, strain and sex, key methods, principal findings, and study conclusions.	1
Background	#12a	Include sufficient scientific background to understand the rationale and context for the study, and explain the experimental approach.	1
Background	#12b	Explain how the animal species and model used address the scientific objectives and, where appropriate, the relevance to human biology.	1
Objectives	#13	Clearly describe the research question, research objectives and, where appropriate, specific hypotheses being tested.	2
Ethical statement	#14	Provide the name of the ethical review committee or equivalent that has approved the use of animals in this study and any relevant licence or protocol numbers (if applicable). If ethical approval was not sought or granted, provide a justification.	2
Housing and husbandry	#15	Provide details of housing and husbandry conditions, including any environmental enrichment.	2
Animal care and monitoring	#16a	Describe any interventions or steps taken in the experimental protocols to reduce pain, suffering, and distress.	2
Animal care and monitoring	#16b	Report any expected or unexpected adverse events.	2
Animal care and monitoring	#16c	Describe the humane endpoints established for the study, the signs that were monitored, and the frequency of monitoring. If the study did not set humane endpoints, state this.	2
Interpretation/scientific implications	#17a	Interpret the results, taking into account the study objectives and hypotheses, current theory, and other relevant studies in the literature.	7
Interpretation/scientific implications	#17b	Comment on the study limitations, including potential sources of bias, limitations of the animal model, and imprecision associated with the results.	7
Generalisability/translation	#18	Comment on whether, and how, the findings of this study are likely to generalise to other species or experimental conditions, including any relevance to human biology (where appropriate).	7
Protocol registration	#19	Provide a statement indicating whether a protocol (including the research question, key design features, and analysis plan) was prepared before the study, and if and where this protocol was registered.	2
Data access	#20	Provide a statement describing if and where study data are available.	
Declaration of interests	#21a	Declare any potential conflicts of interest, including financial and nonfinancial. If none exist, this should be stated.	1
Declaration of interests	#21b	List all funding sources (including grant identifier) and the role of the funder(s) in the design, analysis, and reporting of the study.	1
